# Acute compartment syndrome of the leg due to infection following an insect bite: A case report

**DOI:** 10.1097/MD.0000000000011613

**Published:** 2018-07-27

**Authors:** Jianzhang Wang, Qiang Duan, Xiaolong Sun, Xiang Mou, Baoqiang Song, Hua Yuan

**Affiliations:** a Department of Plastic and Reconstructive Surgery; b Department of Rehabilitation Medicine, Xijing Hospital, Fourth Military Medical University, Xi’an, China.

**Keywords:** compartment syndrome, debridement, fasciotomy, insect bite, rehabilitation

## Abstract

**Rationale:**

Acute compartment syndrome is a highly aggressive condition, which needs rapid diagnosis and surgical emergency. Most cases are caused by trauma, fractures, surgeries, or vascular injury, while other causes are easily misdiagnosed.

**Patients concerns:**

A 29-year-old female, with a medical history of an insect bite on the left calf but not recent trauma, was admitted to the hospital due to the swelling and pain around the bite area.

**Diagnoses:**

Acute compartment syndrome of the lower leg.

**Interventions:**

After admission, she developed septic shock symptoms, given intravenous antibiotics treatments. However, the condition worsened with increasing pain, loss of sensation, tense swelling, and severe pain to any stretch of the tissues. Thus the patient received fasciotomy followed by repeat and thorough debridement. After the wounds healed completely, systematic rehabilitation was performed for three weeks.

**Outcomes:**

After three months of follow-up, the patient is able to walk, and moves up and down the stairs, independently.

**Lessons:**

Our case highlights the possibility of acute compartment syndrome caused by insect bites when the patient presents with the signs of the condition, and the importance of earlier rehabilitation interventions to improve the functional outcome post operation.

## 1 Introduction

Compartment syndrome is a condition in which the circulation is compromised by increased pressure within a space.^[1]^ If not treated promptly, it can progress to a series of sequelae including: weakness, contracture, deformity, motor paralysis, and sensory neuropathy.^[2]^ Therefore, timely diagnosis and surgical decompression are the most effective methods to prevent the morbid consequences.^[3]^

Most compartment syndromes are complicated with trauma, fractures, surgeries, or vascular injury.^[4]^ Infection secondary to insect bites with infectious cellulitis and myositis is a rarely known cause of compartment syndrome. Although insect bites are common, life-threatening sequelae are rare if excluding anaphylaxis.^[5]^ So people tend to ignore the possible secondary infection when bit by insects. Here, we reported a case of compartment syndrome in the calf due to the infection secondary to an insect bite. The purpose of this case is to demonstrate a rare, but serious, complication of insect bites and to emphasize the importance of early fasciotomy, repeat and thorough debridement, and postoperative rehabilitation to decrease the severe sequelae of compartment syndrome.

## 2 Case report

A 29-year-old woman who had been bit by an insect on the left calf was admitted to our hospital with a chief complaint of continuous painful swelling of the bit area for 3 days. After scratched, the bite area became red and inflamed. The injury was not considered severe by the patient initially and the swelling of the calf was treated by self-medication with heat-clearing and detoxifying effects. The aggravating swelling and pain of the left calf impelled her to seek medical advice. After admission to our hospital, the patient developed septic shock symptoms characterized with diminished consciousness, pale skin, hypothermia, lack of urine output, and undetectable blood pressure. Laboratory studies revealed a white blood count of 13.8 × 10^9^ cells/L, neutrophil count of 12.24 × 10^9^ cells/L, and 88.7% polymorphonuclear neutrophils. She was admitted to the intensive care unit, receiving intravenous fluids and broad spectrum antibiotics treatments. Besides, she denied any history of diabetes mellitus, alcoholism, liver diseases, or trauma.

**Figure 1:**
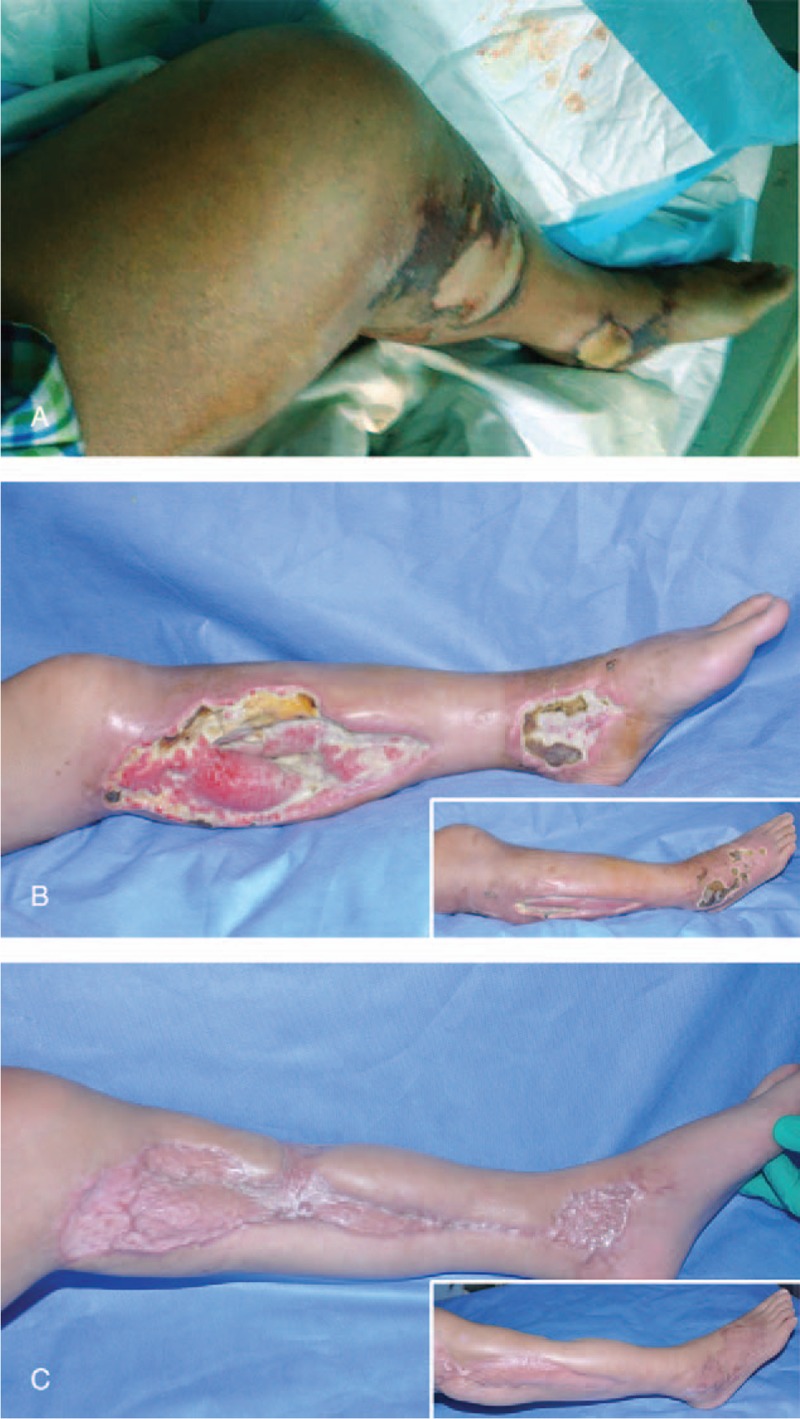
Clinical appearance of the left calf before fasciotomy (A), on day 10 post the first fasciotomy and debridement (B), and after the wounds healed (C). The inserted photos in (B) and (C) showed the outside of the left calf.

In the intensive care unit, the swelling increased and extended proximally to left knee and foot, complicated with blisters (Fig. 1A). Additionally, the patient developed cutaneous necrosis in the left ankle and popliteal space. Clinical examination showed her entire left calf was tensely swollen both medially and laterally, and the most obvious pain was localized to the bit area of the left calf. She had a loss of superficial touch sensation and 2-point discrimination over the entire sole of the left calf. She was unable to move her left leg actively, and any passive movements of the left calf, knee, and ankle joints caused severe pain. Palpation of the whole left leg revealed a mildly increased skin temperature and exquisite pain compared with her contralateral leg. The main differential diagnose was from deep vein thrombosis (DVT). Subsequent venous Doppler ultrasonography found no evidence of DVT, and only subcutaneous edema at the lower leg. On the basis of the postmedical history and clinical findings, in particular, increasing pain, loss of sensation, tense swelling, and severe pain to any stretch of the tissues, we diagnosed acute compartment syndrome affecting her left leg.

According to the diagnosis, the patient was then transferred to the operating room for surgical inspection of the tissues and decompression of the compartments by fasciotomy. A medial incision was made 2 cm posterior to the posteromedial border of the tibia. Extensive purulence was encountered in the subcutaneous fat and superficial fascia, which confirmed the diagnosis of infectious cellulitis. The purulence was collected for aerobic and anaerobic bacterial culture. After fascia was incised, the deep layer of gastrocnemius and partial soleus were found dark red, with widespread necrosis as well as drainage of thick and amber pus, which confirmed the diagnosis of infectious myositis. Next, the dissection proceeded to transverse intermuscular septum over flexor hallucis longus muscle, flexor digitorum longus muscle, and tibialis posterior muscle to release the deep posterior compartment. The necrotic muscle and the grossly infected soft tissue were thoroughly debrided without the injury of posterior tibial artery, veins, and tibial nerve. Then, the lateral incision was made 3 cm lateral to the crest of the tibia. Layer dissection through this incision to the periosteum revealed a little purulence but no necrotic muscle. The fascia over the anterior and lateral compartments was completely released. After inspection, decompression, and debridement, the areas were irrigated sequentially with 3% perhydrol, diluent iodophor, and normal saline. Eventually, the wounds were closed by vacuum sealing drainage to improve the wound circumstance and further reduce intracompartment pressures. She was treated with elevation of the left leg, empirical antibiotic therapy, and daily monitoring of peripheral blood circulation. Considering the severity of the left calf, we closely monitored the function of renal and prepared to amputate the infected limb in case of possible renal failure and death due to acute rhabdomyolysis.

On day 3 postoperation, the patient reported a remarkable pain relief in the area of the incisions. A reduction of the edema was also found. Cefoperazone–Sulbactam was treated intravenously twice a day on the basis of bacteria culture revealing heavy infection of *Staphylococcus aureus* and *Escherichia coli*. On day 10 postoperation, vacuum sealing drainage was removed, and the surgical site was still riddled with necrotic and purulent tissue (Fig. 1B). Additionally, the skin of former swelling popliteal fossa and foot were ulcerated and it was thought to arise from the spread of infection. To reduce the severity of deep wound infection, thorough debridement was performed on day 12. We explored and found purulence and necrosis bestrewed with segmental posterior muscular group of calf, especially the fascial spaces. Different from calf, wounds in foot and popliteal fossa just deepened to subcutaneous tissue. Then the necrotic, injected tissue, and inflammatory granulation were completely excised until healthy, and bleeding tissue was exposed. Eventually, the wounds, especially the deep muscle tissues, were irrigated repeatedly with 3% perhydrol, diluent iodophor, and normal saline, and were closed by vacuum sealing drainage. Scheduled redebridements were performed every 7 to 8 days until all necrotic and injection tissues were removed. When the wounds were well granulated, we applied an anterolateral thigh flap transplantation to close the medial and lateral calf wounds and a split-thickness skin gift to close the medial and lateral foot. After 6 weeks, medial and lateral calf wounds healed well (Fig. 1C).

When the wound healed completely, the patient underwent systematic rehabilitation for approximately 3 weeks, including rehabilitation assessment and treatment. The rehabilitation assessment was performed firstly. As shown in Table 1, the main problems were the ability barrier of daily life activities (standing, walking, transfer, lavatory, stairs, etc) and the movement dysfunction of the left knee and ankle (strength, mobility, etc). Besides, the sensory of the left lower extremity below the knee, the motor and sensory nerve conduction function of the left tibial nerve, and peroneal nerves were partly impaired. So the following rehabilitation programs were drew up: the low-power helium-neon laser to promote wound healing^[6]^; the ultrasound and audio pulse therapy to soften the scar and loosen the adhesion; the wax, arthrosis, and cold therapy to improve the motion of the joints. Also, the functional transcranial magnetic stimulation (10 Hz trains for 2 seconds; repeated 70 times with an inter-train interval of 4 seconds, a total of 1400 pulses and 7 minutes) was used to promote nerve function recovery. All the above treatments were performed daily. After 3 weeks, the patient underwent a second rehabilitation evaluation (Table 1). All the scores of the evaluation index increased, albeit limited. Considering the high costs for the continued in-hospital rehabilitation, the patient chose to wear an orthopedic insole to achieve standing and then returned home. During the following 3 months after discharge, we conducted a telephone follow-up for the patient. After 1 month, she was able to walk independently, but slowly and instability. Moving up and down the stairs was limited. Three months later, the activities of daily living are markedly improved. She is able to walk, and moves up and down the stairs, independently.

**Table 1:**
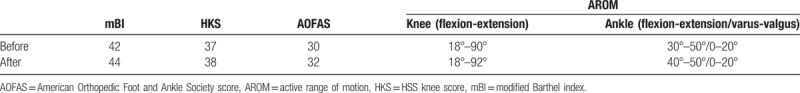
The change of functional assessment of the left leg before and after rehabilitation.

## 3 Discussion

Compartment syndrome occurs when intracompartmental pressure is increased, leading to tissue ischemia and function disorder of the involved muscle, nerve, and vascular. Commonly, compartment syndrome occurs after high-energy trauma, especially crush injury.^[7]^ Other causes include burns, animal bites, bleeding disorders, and tight circumferential dressings.^[8]^ Compared with high-energy trauma, infection caused by insect bites is considered rare, thus with the risk of being overlooked. It is important to be aware of the patient's medical histories when considering these unusual causes of compartment syndrome. To our knowledge, Evans et al^[9]^ firstly described a case of compartment syndrome caused by a streptococcal cellulitis following an insect bite, which reminded us of a possible serious complication of insect bites.

Pain out of proportion, pain with passive range of motion, pallor, paresthesia, paralysis, and pulselessness are known as the “Ps” of compartment syndrome.^[10]^ Among them, pain out of proportion and pain with passive range of motion have been referred to the most important and sensitive symptoms.^[11]^ In this case, the presence of severe pain was out of proportion to the clinical appearance of the left calf. Also, the pain was aggravated by passive movements of the left calf. Moreover, the tense swelling of both medial and lateral calf, the loss of sensations in the involved place, the history of insect bites, and clinical course of septic shock further supported the diagnosis of compartment syndrome. We did not measure the interstitial tissue pressures because: the diagnosis of acute compartment syndrome is primarily determined by the typical clinical manifestations and history. Also, there is no consensus recommendations on what pressure thresholds were best used for fasciotomy.^[12,13]^

Given the severe consequences of compartment syndrome, fasciotomy as well as early diagnosis is critical to improve the clinical outcome of patients. In this case, the prompt fasciotomy saved the patient's lower leg, further emphasizing the importance of the rapid appreciation of the condition and definitive treatment. Multiple surgical approaches have been developed, including “double incisions” and “single incision.”^[14,15]^ Considering infection had spread over a large area and led to increased pressure of multiple fascia space, especially the posterior compartment, we chose the former one. However, in spite of debridement, the deep tissues and muscles still suffered serious damage due to the infectious cellulitis and myositis. Previous studies have shown that debridement and irrigation with appropriate antibiotics are curative for deep tissue infection, especially in the immediate postoperative period.^[16]^ We performed repeat and thorough debridement, to remove the infection and necrotic tissue, and adjusted sensitive antibiotic based on the change of bacterial culture. From our clinical experience, through this comprehensive way, we salvaged the involved limb as much as possible.

A previous meta-analysis has suggested that postoperative rehabilitation may be beneficial to avoid repeat surgical intervention for the patients with exertional compartment syndrome.^[17]^ The functional assessment for the left leg of our patient was improved after 3 weeks rehabilitation. Also, No additional surgical intervention was performed during the rehabilitation and the follow-up period. However, it should be noted that the rehabilitation effects are limited. There are 2 possible reasons: The rehabilitation intervention was performed 6 months after the onset. At this stage, the injured tissue had passed the acute phase of inflammation, edema, and reached the stage of scar formation. Softening scar physical treatment is less effective in this particular period. (2) For the patients who develop compartment syndrome arising from infection, muscles, and nerves are more susceptible to damage due to the rapid spread of the infection. Thus, many muscles and tendons had to be removed, which resulted in much reduction in the lower muscle strength and prolonged contracture of the joint capsule. Besides, the electromyogram (EMG) examination showed that the left lower tibial nerve, peroneal nerve, and sural nerve of the patient were damaged partially, which further affected the function of muscles and the proprioception. So, we finally adopted the function compensatory to maximize the patient's daily life activities, and the follow-up results were satisfactory. From this case, we should emphasize the importance of early rehabilitation intervention, such as earlier knee and ankle function placement, earlier postoperative effective isotonic/isometric muscle training, and passive joint activity. All these earlier rehabilitation interventions can effectively avoid or reduce the occurrence of postoperative muscle atrophy, tendon shortening, and joint contracture, which provides a better condition for the recovery of postoperative function, and reduce the total cost of rehabilitation for the patient.

## 4 Conclusion

Compartment syndrome is a rare, but serious, complication of infection due to insect bites. The diagnosis should be based on the clinical examinations, medical history, and clinical course. Prompt fasciotomy, repeat and thorough debridement, and appropriate antibiotic therapy are the most effective methods to prevent amputation and morbid consequences. Notably, this case also showed a limit of rehabilitation effect possibly due to a serious local damage, a longer course of disease, and a late intervention, suggesting the significance of earlier rehabilitation interventions.

### 4.1 Consent

Written, informed consent was obtained from the patient to use the content and imaging material for publication.

## Author contributions

Acquisition of data: Jiangzhang Wang, Qiang Duan, Xiaolong Sun. Analysis and interpretation of data: Jiangzhang Wang, Qiang Duan, Xiang Mou, Baoqiang Song. Drafting of the manuscript: Jiangzhang Wang, Qiang Duan, Xiaolong Sun. Critical revision of the manuscript for important intellectual content: Hua Yuan, Xiaolong Sun. All authors read and approved the final manuscript.

**Data curation:** Jianzhang Wang, Qiang Duan, Xiaolong Sun.

**Formal analysis:** Qiang Duan, Xiaolong Sun, Xiang Mou, Baoqiang Song.

**Supervision:** Hua Yuan.

**Writing – original draft:** Qiang Duan, Xiaolong Sun.

**Writing – review & editing:** Xiaolong Sun, Hua Yuan.
